# Liver transplantation for hepatocellular carcinoma using grafts from uncontrolled circulatory death donation

**DOI:** 10.1038/s41598-021-92976-5

**Published:** 2021-06-29

**Authors:** Anisa Nutu, Iago Justo, Alberto Marcacuzco, Óscar Caso, Alejandro Manrique, Jorge Calvo, Álvaro García-Sesma, María García-Conde, María Santos Gallego, Carlos Jiménez-Romero

**Affiliations:** 1 Unit of HPB Surgery and Abdominal Organ Transplantation, “Doce de Octubre” University Hospital. Instituto de Investigación (imas12), Department of Surgery, Faculty of Medicine, 4ª Planta. Ctra Andalucía Km 5,4, 28041 Madrid, Spain; 2 Service of Radiology, “Doce de Octubre” University Hospital, Madrid, Spain

**Keywords:** Cancer, Surgical oncology

## Abstract

Controversy exists regarding whether the rate of hepatocellular carcinoma (HCC) recurrence after orthotopic liver transplantation (OLT) differs when using livers from donation after controlled circulatory death (DCD) versus livers from donation after brain death (DBD). The aim of this cohort study was to analyze rates of HCC recurrence, patient survival, and graft survival after OLT for HCC, comparing recipients of DBD livers (n = 103) with recipients of uncontrolled DCD livers (uDCD; n = 41). No significant differences in tumor size, tumor number, serum alpha-fetoprotein, proportion of patients within Milan criteria, or pre-OLT bridging therapies were identified between groups, although the waitlist period was significantly shorter in the uDCD group (*p* = 0.040). HCC recurrence was similar between groups. Patient survival was similar between groups, but graft survival was lower in the uDCD group. Multivariate analysis identified recipient age (*p* = 0.031), pre-OLT bridging therapy (*p* = 0.024), and HCC recurrence (*p* = 0.048) as independent risk factors for patient survival and pre-OLT transarterial chemoembolization (*p* = 0.045) as the single risk factor for HCC recurrence. In conclusion, similar patient survival and lower graft survival were observed in the uDCD group. However, the use of uDCD livers appears to be justified due to a shorter waitlist time, and lower waitlist dropout and HCC recurrence rates.

## Introduction

Hepatocellular carcinoma (HCC) represents the sixth most common malignancy and the third leading cause of cancer-related deaths around the world^1,2^. It is frequently diagnosed incidentally or during screening programs, as people who develop HCC typically do not manifest symptoms until the tumor has reached a late stage.

Orthotopic liver transplantation (OLT) is considered the treatment of choice for patients with early-stage HCC (Milan criteria). In Spain, 1,227 patients underwent OLT during 2019, with 282 (23%) receiving transplants for HCC^3^. However, the number of available liver grafts remains insufficient to treat all patients who require an OLT for malignant or benign disease. To increase the liver graft pool and thereby decrease waitlist mortality, new strategies have been developed, such as the use of livers from marginal donors^4^, including livers donated after controlled circulatory death (cDCD)^5,6^, uncontrolled circulatory death (uDCD)^7–11^, and older donors^12,13^. Recently, liver grafts with major extended criteria (steatosis > 40%, age > 65 years, and prolonged cold ischemia time [CIT]) have been used in recipients with HCC without impairing patient survival or HCC recurrence^14^.

In Type 2 uDCD donation, the donor sustained a witnessed out-of-hospital cardiac arrest and underwent unsuccessful cardiopulmonary resuscitation, whereas in type 3 cDCD donation, organs are recovered after death confirmation from patients with irreversible brain injury or respiratory failure, in whom life-sustaining treatment has been withdrawn^15^. Liver grafts from both uDCD and cDCD donation are subjected to longer warm ischemia periods, compared to grafts donated after brain death (DBD), which results in a higher likelihood of ischemia/reperfusion injury (IRI). Use of uDCD livers has been associated with higher rates of primary nonfunction (PNF) and biliary complications (BCs) than OLT with cDCD and DBD donors. This has been attributed to the longer period of ischemia with uDCD, which is the sum of the donor circulatory arrest time, duration of cardiopulmonary resuscitation (CPR), duration of normothermic regional perfusion (NRP), and recipient warm ischemia time (WIT)^7,11^. However, use of uDCD livers may be justified given the length of time OLT candidates remain on the waitlist (mean, 124 days)^3^, and the associated dropout risk.

As patients with HCC often exhibit compensated, clinically stable disease with a relatively low Model for End-stage Liver Disease (MELD) score when placed on the waitlist, these individuals are usually ideal candidates for cDCD OLT, as they are able to tolerate potential complications related to the use of marginal livers. Nevertheless, several reports have demonstrated increased HCC recurrence after OLT using cDCD, as well as reduced patient and graft survival^16–18^. These results were explained in a mouse model, which showed that IRI is a strong stimulus for recurrent intrahepatic tumor growth in transplanted liver ^16,18,19^.

The aim of this study was to analyze use of liver grafts from uDCD donation in patients with HCC, comparing rates of patient and graft survival, HCC recurrence, and recurrence-free survival with a control group of patients with HCC who received livers from DBD donation.

## Patients and methods

### Study population and study design

Between April 1986 and December 2016, 1,876 OLTs were performed in adults and children at our institution. From January 2006 to December 2016, 75 of these OLTs were performed using livers from uDCD donation. This retrospective cohort study compared 103 adults with HCC who underwent OLT using liver grafts from DBD donation (DBD Group) with 41 adults with HCC who underwent OLT with livers from uDCD donation (uDCD Group). There was a chronological correlation between cases and controls. This study was performed in accordance with the ethical guidelines of the declaration of Helsinki, and was approved by Institutional Review Board/Ethics Committee of “12 de Octubre” University Hospital. The organs were not procured from prisoners. The study was closed on October 31, 2018, after a minimum follow-up of 22 months after OLT.

### Criteria for acceptance of uDCD and DBD liver grafts

All liver donors in this study were maintained with NRP for a maximum of 300 min before initiation of organ perfusion, in accordance with our previously described protocol for uDCD donors^11^. The criteria for acceptance of uDCD livers were as follows: donor age between 14 and 55 years; maximum transaminase levels < 4 times the upper limit of normal; and absence of alcoholic disease, drug addiction, history of cancer, hepatitis B and/or C infection, HIV infection, violent death, or abdominal trauma. Additional criteria, which were assessed at the time of organ procurement, included good appearance, consistency, and vascularization of the liver graft, and no evidence of ischemia of the gallbladder, common bile duct, or intestines.

Graft donor warm ischemia time (DWIT), also called pre-NRP WIT, was defined as the sum of circulatory arrest time and duration of pre-NRP CPR, whereas recipient WIT (RWIT) was defined as the interval from removal from cold preservation solution to completion of portal vein anastomosis. We routinely performed liver graft biopsy before cold perfusion and discarded grafts with fibrosis or > 30% macrosteatosis. We also discarded grafts when DWIT or RWIT exceeded the times established in the protocol. Once it is established that the liver graft from uDCD meets the criteria for OLT, the liver transplant team selects a recipient from their own waitlist that has previously signed the informed consent. This restriction (i.e. only offering the graft to the liver transplant team that has performed the procurement) was set in order to minimize ischemia time. In our hospital we have a single transplant waitlist that includes both patients that have only accepted livers from DBD and those who accept both donation types (DBD/uDCD).

### Inclusion and exclusion criteria, candidate information, and transplant technique

The inclusion criteria for OLT recipients were age > 18 years, HCC as the main indication for transplant, and within the Milan^20^ or University of California, San Francisco (UCSF) criteria for transplantation^21^. We excluded patients who received partial or combined transplants, underwent retransplantation, were positive for HIV, underwent OLT for fulminant hepatitis, or received grafts from donors > 70 years of age (for DBD recipients). Use of uDCD livers was avoided in patients with previous abdominal surgery or a MELD score > 30. No exception points were added to the MELD score for patients with tumors > 2 cm in diameter. To prevent tumor progression, transarterial chemoembolization (TACE) was usually performed in patients with several tumors or a single tumor and ascites, whereas radiofrequency ablation was generally used in patients with a single tumor. Recipient hepatectomy was performed using the vena cava sparing technique (piggy-back). In most cases, biliary reconstruction was performed as a cholechocholedochostomy without T-tube.

### Comparisons of donor and pre-OLT recipient variables

Donor variables common to both groups were compared between groups. These included age, sex, body mass index (BMI), cause of death, vasopressor use, transfusion, intensive care unit (ICU) stay, cardiac arrest, steatosis, cold ischemia time (CIT), and RWIT. The following pre-OLT recipient characteristics were also compared between groups: age, sex, BMI, OLT indication, MELD score, comorbidities, waitlist duration, and laboratory values. Likewise, a number of perioperative variables were compared between groups, including biliary reconstruction, transfusion of blood products, post-OLT liver function, immunosuppression, retransplantation, post-OLT complications, patient and graft survival, and recurrence-free survival. Pre- and post-OLT tumor characteristics and HCC recurrence were also compared between groups.

PNF was defined as severe clinical deterioration requiring retransplantation or progressing to death, which was the consequence of irreversible liver graft failure within 10 days after OLT, in the absence of vascular complications^22^. Among BCs, non-anastomotic biliary strictures (NABS) were defined as any stricture, dilation, or irregularity of the intrahepatic or extrahepatic bile ducts of the liver (hilum), whereas anastomotic biliary strictures (ABS) were defined as lesions localized to the anastomosis site^23^.

### Immunosuppression

The immunosuppressive regimen consisted of tacrolimus and prednisone. Corticosteroids were usually discontinued between 3 and 6 months after OLT. Tacrolimus trough levels were maintained between 10–15 ng/mL during the 1st months after transplantation, between 8 and 12 ng/mL until the 6th month, and between 5 and 8 ng/mL thereafter. Mild acute rejection was treated with increasing the dose of tacrolimus, whereas moderate or severe episodes was treated with 1 g methylprednisolone intravenously for 3 days.

Currently, we use a tacrolimus-based regimen with lower tacrolimus doses, which includes mycophenolate mofetil (MMF) or a mammalian target of rapamycin inhibitor (mTORi) in the presence of renal dysfunction, hypertension, diabetes, de novo tumor, or HCC as OLT indication. Conversion from tacrolimus to MMF or mTORi monotherapy is performed on long-term follow-up in recipients who undergo OLT for HCC or in patients with severe adverse tacrolimus effects but stable liver function.

### Statistical analysis

Quantitative variables were expressed as mean and standard deviation or median and interquartile (25%–75%) range. Qualitative variables were expressed as percentages. Differences between qualitative variables were assessed by chi-square test or Fisher's exact test, as appropriate. Quantitative variables were compared using *t*-test or Mann–Whitney U test, depending on whether the data were normally distributed. Graft and patient survival rates were estimated using the Kaplan–Meier method, and survival curves were compared using the log-rank test. Donor and recipient variables with *p* values < 0.10 in univariate analysis were subsequently investigated in multivariate analysis using Cox's regression model to evaluate associations between baseline variables and patient or graft survival. Results were expressed as hazard ratios (HRs) and 95% confidence intervals (CIs). *P* values < 0.05 were considered statistically significant. All analyses were performed with SPSS Statistics Version 24.

### Ethical approval

This study was approved by our Institutional Review Board.

### Informed consent

All patients included in this study firmed inform consent to treatment.

## Results

### Donor and recipient characteristics

uDCD donors were significantly younger than DBD donors (41.0 ± 10.0 y vs. 48.0 ± 13.0 y; *p* = 0.001). Mean BMI, as well as rates of cardiac arrest and vasopressor use, was significantly higher in the uDCD group. There were no statistically significant differences between groups for CIT, RWIT, and rates of donor microsteatosis or macrosteatosis.

During the period of this study, a total of 11 (7.6%) patients dropped out of the waitlist: 9 (6.2%) patients who had only signed the informed consent for DBD livers versus 2 (1.4%) patients who had signed the informed consent for both DBD and uDCD livers (p = 0.366). The dropout causes for patients in the DBD group were death in 6 patients, and tumor progression in 3; whereas in the DBD and uDCD group the causes were death in one, and tumor progression in the other patient.

Mean age was significantly higher in uDCD recipients than in DBD recipients (57.0 ± 8.1 y vs. 61.0 ± 6.6 y; *p* = 0.007). The prevalence of other OLT indications associated with HCC was similar in both groups, as was the prevalence of other morbidities. The median MELD score was significantly higher in recipients of uDCD donors (*p* = 0.008). Among laboratory variables, recipients of uDCD livers had significantly lower platelet counts and prothrombin rates (Table 1).

**Table 1:** Donor and pre-liver transplantation recipient characteristics.

	DBD group	uDCD group	*p* value
	(n = 103)	(n = 41)	
**Donor characteristics**			
Mean age (y)	48 ± 13	41 ± 10	*0.001*
**Sex**			
Male	68 (66%)	39 (95.1%)	*0.004*
Female	35 (34%)	2 (4.9%)	*0.004*
BMI (kg/m^2^)	24.2 ± 5.1	27.1 ± 5.4	*0.040*
Cardiac arrest	38 (39.6%)	41 (100%)	*0.001*
Vasopressor use	61 (59.2%)	41 (100%)	*0.001*
PRBC transfusion	25 (24.2%)	2 (4.8%)	0.089
**Steatosis**			
No steatosis	54 (52.8%)	25 (60%)	0.500
Microsteatosis	11 (10.7%)	3 (7.3%)	0.604
Macrosteatosis < 30%	35 (34%)	12 (29.2%)	0.604
Cold ischemia time (min)	383 ± 156	371 ± 100	0.740
Recipient WIT (min)	60 (55–75)	60 (50–75)	0.121
**Recipient characteristics**			
Mean age (y)	57 ± 8.1	61 ± 6.6	*0.007*
**Sex**			
Male	87 (84.5%)	33 (80.5%)	0.361
Female	16 (15.5%)	8 (19.5%)	0.361
BMI (kg/m^2^)	28.5 ± 4.4	28.3 ± 4.6	0.804
**OLT indications**			
HCV cirrhosis	70 (67.9%)	30 (73.1%)	0.344
HBV cirrhosis	16 (15.5%)	3 (7.3%)	0.148
Alcoholic cirrhosis	33 (32.1%)	11 (26.85)	0.344
Other diseases	2 (1.9%)	1 (2.4%)	0.492
MELD score	10 (8–14)	13 (9.5–18.5)	*0.008*
**Comorbidities**			
Cardiac	11 (10.6%)	7 (17.1%)	0.302
Respiratory	9 (8.7%)	2 (4.8%)	0.302
Kidney	6 (5.8%)	4 (9.7%)	0.302
**Laboratory values (pre-OLT)**			
Hemoglobin (g/dL)	12.6 ± 2.1	12.4 ± 2.3	0.618
Platelets (n × 10^3^/mm^3^)	95 ± 49	76 ± 32	*0.020*
Prothrombin rate (%)	75 ± 18	63 ± 18	*0.001*
Bilirubin (mg/dL)	1.2 (0.6–2.7)	2.2 (1.3–3)	0.748
Serum creatinine (mg/dL)	0.8 (0.6–0.9)	0.8 (0.7–0.9)	0.767
Serum albumin (g/L)	3.6 (3–4.1)	3.2 (2.6–3.7)	0.215

Italic values indicate statistically significant.

*AFP* alpha-fetoprotein; *BMI* body mass index; *DBD* donation after brain death; *HBV* hepatitis B virus; *HCV* hepatitis C virus; *MELD* model for end stage liver disease; *OLT* orthotopic liver transplantation; *PRBC* packed red blood cells; *uDCD* uncontrolled donation after circulatory death; *WIT* warm ischemia time.

### Perioperative characteristics and post-OLT complications

Types of biliary reconstruction techniques were similarly distributed in the two groups, whereas the volume of all intraoperative blood products was significantly higher in uDCD recipients. Liver function parameters were similar between groups on the 7^th^ post-OLT day, but median gamma-glutamyl transpeptidase (GGT) and bilirubin were significantly lower in uDCD recipients on the 30th post-OLT day. Immunosuppressor use, hospital stay, or ICU stay did not differ significantly between groups.

Regarding post-OLT complications, the rate of PNF was significantly higher in uDCD recipients (4 cases; 9.7%) than in DBD recipients (1 case; 1.0%) (*p* = 0.023). Hepatic artery thrombosis occurred in 2 (1.9%) recipients in the DBD group and 3 (7.3%) patients in the uDCD group (*p* = 0.150). Fewer patients (9; 8.7%) developed BCs in the DBD group than in the uDCD group (10; 24.4%), but this finding did not reach statistical significance (*p* = 0.071). Rates of acute and chronic rejection were similar in both groups.

The rate of retransplantation was significantly higher in recipients of uDCD livers (5 patients; 12.2%) than in recipients of DBD livers (1 patient; 1.0%; *p* = 0.01). Reasons for retransplantation were PNF and NABS (4 patients and 1 patient, respectively) in the uDCD group. For the 1 patient who underwent retransplantation in the DBD group, PNF was the indication (Table 2).

**Table 2:** Perioperative characteristics and post-orthotopic complications.

	DBD Group	uDCD Group	*p* value
	(n = 103)	(n = 41)	
*Perioperative variables*			
**Biliary reconstruction**			
Chol-Chol without T tube	95 (92%)	38 (92%)	0.551
Chol-Chol with T tube	8 (8%)	3 (8%)	0.551
Transfusion			
PRBC (units)	5.7 ± 7	13.1 ± 15	*0.002*
FFP (units)	7.9 ± 6.9	16.5 ± 14.1	*0.001*
Platelets (units)	1.4 ± 1	2.6 ± 1.9	*0.002*
Fibrinogen (units)	1.2 ± 2.1	2.7 ± 3.5	*0.020*
**Post-OLT liver function (7th d)**			
GOT (IU/L)	48 (33–78)	37 (22–63)	0.388
GPT (IU/L)	194 (117–307)	165 (109–283)	0.461
GGT (IU/L)	266 (158–431)	191 (117–464)	0.149
Bilirubin (mg/dL)	1.1 (0.7–1.5)	0.9 (0.6–2.7)	0.461
**Post-OLT liver functions (30th d)**			
GOT (IU/L)	31 (18–54)	17 (14–40)	0.112
GPT (IU/L)	58 (30–118)	30([20–77)	0.099
GGT (IU/L)	133 (82–283)	61 (56–202)	*0.020*
Bilirubin (mg/dL)	0.8 (0.5–1.1)	0.6 (0.4–0.8)	*0.034*
**Immunosuppression (1-yr post-OLT)**			
Tacrolimus	82 (79.6%)	35 (85.3%)	0.367
Cyclosporine	1 (1%)	0	0.754
Mycophenolate mofetil	56 (54.3%)	25 (61%)	0.368
mTORi	21 (21.4%)	6 (14.7%)	0.567
ICU stay (d)	3 (2–5.5)	4 (2.5–8)	0.145
Hospital stay (d)	15 (12–21)	19 (12–26.5)	0.802
**Post-OLT complications**			
Primary non-function	1 (1%)	4 (9.7%)	*0.023*
Hepatic artery thrombosis	2 (1.9%)	3 (7.3%)	0.150
Biliary complications	9 (8.7%)	10 (24.4%)	0.071
NABS	4 (3.8%)	5 (12.2%)	–
ABS	5 (4.8%)	5 (12.2%)	–
Acute rejection	37 (35.9%)	13 (31.7%)	0.245
Chronic rejection	3 (2.9%)	1 (2.4%)	0.664
Liver retransplantation	1 (1%)	5 (12.2%)	*0.010*
Primary non-function	1 (1%)	4 (9.7%)	*0.023*
NABS	0	1 (2.4%)	0.408

Italic values indicate statistically significant.

*ABS* anastomotic biliary stenosis; *BMI* body mass index; *Chol-Chol* choledochocholedochostomy; *DBD* donation after brain death; *FFP* fresh frozen plasma; *GGT* gamma-glutamyl transpeptidase; *GOT* glutamic oxaloacetic transaminase; *GPT* glutamic pyruvic transaminase; *HBV* hepatitis B virus; *HCV* hepatitis C virus; *ICU* intensive care unit; *mTOR* mammalian target of rapamycin inhibitors; *NABS* non-anastomotic biliary stenosis; *OLT* orthotopic liver transplantation; *PRBC* packed red blood cells; *uDCD* uncontrolled donation after circulatory death.

### Pre-OLT and post-OLT tumor characteristics and post-OLT recurrence

Pre-OLT and post-OLT HCC characteristics (histological examination of the explant liver), as well as post-OLT HCC recurrence data, are depicted in Table 3. There were no statistically significant differences in mean tumor size or mean tumor number between recipients of DBD and uDCD livers. Alpha-fetoprotein (AFP) levels at the time of placement on the waitlist were not significantly different between the two groups. Similarly, we detected no significant differences in the percentage of patients in both groups within Milan criteria (86% in DBD vs. 95.1% in uDCD; *p* = 0.200) or UCSF criteria at the time of waitlist inclusion (16.6% in DBD vs. 4.9% in uDCD; *p* = 0.351). The time from HCC diagnosis to OLT was significantly longer in DBD recipients than in uDCD recipients (19.1 ± 7.1 mo vs. 12.3 ± 7.1 mo; *p* = 0.038). The time from OLT waitlist inclusion to OLT was also significantly longer in the DBD group than in the uDCD group (7.9 ± 5.4 mo vs. 5.3 ± 3.0 mo; *p* = 0.040). Similar proportions of patients in both groups (48.5% in DBD and 48.7% in uDCD; *p* = 0.413) received pre-OLT HCC bridging therapies; rates for pre-OLT TACE or radiofrequency ablation did not differ between groups. After histological examination of the explant liver, we observed no statistically significant differences between groups for median tumor size, number of tumors, and vascular or perineural invasion. The proportion of patients within Milan criteria decreased from pre-OLT to post-OLT (based on liver explant histological findings), although there were no differences between groups either pre- or post-OLT. There were corresponding increases in the percentage of patients within UCSF from pre-OLT to post-OLT, with no significant between-group differences at either time. The proportion of patients exceeding UCSF criteria after OLT were similar in the DBD group (19 patients; 18.4%) and uDCD group (7 patients; 17.1%; *p* = 0.528).

**Table 3:** Preoperative and postoperative tumor characteristics, and post-orthotopic liver transplantation recurrence.

	DBD Group	uDCD Group	*p* value
	(n = 103)	(n = 41)	
**Pre-OLT tumor characteristics**			
Tumor size at waitlisting (cm)	2.7 ± 1.7	3 ± 1.2	0.402
Tumor number at waitlisting	1(1–2)	1 (1–2)	0.402
AFP at waitlisting (ng/mL)	5.1 (3.2–12.6)	13.5 (5.3–54.5)	0.603
Patients within Milan criteria	86 (83.4%)	39 (95.1%)	0.200
Patients within UCSF criteria	17 (16.6%)	2 (4.9%)	0.351
Time from HCC diagnosis to OLT (mo)	19.1 ± 7.1	12.3 ± 7.1	*0.038*
Waitlist duration (mo)	7.9 ± 5.4	5.3 ± 3	*0.040*
Pre-OLT therapy	50 (48.5%)	20 (48.8%)	0.413
TACE	44 (42.7%)	16 (39.0%)	0.841
Radiofrequency	41 (39.8%)	10 (24.4%)	0.210
TACE + radiofrequency	14 (9.7%)	3 (7.3%)	0.395
**Post-OLT tumor characteristics**			
Tumor size (cm)	3.9 (2.1–6.6)	4 (2.5–5.7)	0.373
Tumor number	2 (1–3)	2 (2–3)	0.436
Vascular invasion	16 (15.5%)	3 (7.3%)	0.168
Microvascular invasion	11(10.7%)	3 (7.3%)	0.256
Macrovascular invasion	5 (4.8%)	0	0.144
Perineural invasion	0	1 (2.4%)	0.291
Patients within Milan criteria	61 (59.2%)	27 (65.8%)	0.503
Patients within UCSF	23 (22.3%)	7 (17.1%)	0.440
Patients beyond UCSF	19 (18.4%)	7 (17.1%)	0.528
**Tumor recurrence after OLT**			
Duration of follow-up after OLT (mo)	52 ± 35	56 ± 44	0.550
Time from OLT to recurrence (mo)	33 (3–48)	12 (12–90)	0.091
Recurrence rate	8 (7.8%)	3 (7.3%)	0.597
Liver	2 (1.9%)	2 (4.9%)	0.197
Bones	3 (2.9%)	1 (2.4%)	0.197
Lungs	3 (2.9%)	0 (0%)	0.197
Death from HCC recurrence	4 (3.8%)	1 (2.4%)	0.545

Italic values indicate statistically significant.

*AFP* alpha-fetoprotein; *DBD* donation after brain death; *HCC* hepatocellular carcinoma; *OLT* orthotopic liver transplantation; *TACE* transarterial chemoembolization; *UCSF* University of California, San Francisco; *uDCD* uncontrolled donation after circulatory death.

After a follow-up period of at least 22 months for all patients (mean, 52 ± 35 mo in the DBD group vs. 56 ± 44 mo in the uDCD group; *p* = 0.550), the rate of tumor recurrence was similar in both groups (7.8% in the DBD group vs. 7.3% in the uDCD group; *p* = 0.597). The median time from OLT to tumor recurrence diagnosis was longer in the DBD group than in the uDCD group, although the difference did not reach statistical significance (33 mo vs. 12 mo; *p* = 0.091). Locations of tumor recurrence were the liver, bones, or lungs, which were not significantly different between groups. Four (3.8%) patients died of tumor recurrence in the DBD group, whereas 1 (2.4%) patient died of recurrence in the uDCD group (*p* = 0.545).

### Patient and graft survival and predictive factors

**Figure 1:**
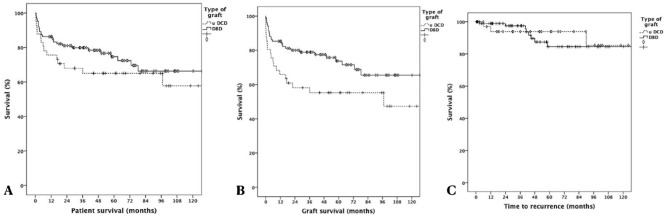
(**A)** Patient survival: 1-, 3- and 5-year were 85%, 78% and 72%, respectively, in DBD recipients and 72%, 65% and 61%, respectively, in uDCD recipients (*p* = 0.249). (**B)** Graft survival: 1-, 3- and 5-year were 84%, 77%, and 71%, respectively, in DBD recipients and 65%, 58% and 58%, respectively, in uDCD recipients (*p* = *0.021*). (**C**) Recurrence-free tumor survival: 1-, 3- and 5-year were 98%, 95% and 82%, respectively, in DBD recipients, and 91%, 79% and 79%, respectively, in uDCD recipients (*p* = 0.754).

Patient and graft survival were lower in the uDCD group, but only graft survival reached a statistically significant difference. Overall, 1-, 3-, and 5-year patient survival rates were 85%, 78%, and 72%, respectively, in DBD recipients and 72%, 65%, and 61%, respectively, in uDCD recipients (*p* = 0.249). The 1-, 3-, and 5-year graft survival rates were 84%, 77%, and 71%, respectively, in DBD recipients and 65%, 58%, and 58%, respectively, in uDCD recipients (*p* = 0.021) (Figs. 1A,B). However, when we excluded PNF cases, graft survival did not differ between groups, with 1-, 3-, and 5-year graft survival rates of 82%, 79%, and 70%, respectively, in DBD recipients and 70%, 62%, and 61%, respectively, in uDCD recipients (*p* = 0.126).

Regarding recurrence-free survival, there were no statistically significant differences between groups. The 1-, 3-, and 5-year recurrence-free survival rates were 98%, 95%, and 82%, respectively, in DBD recipients and 91%, 79%, and 79%, respectively, in uDCD recipients (*p* = 0.754) (Fig. 1C).

On univariate analysis, recipient age, bridging therapies, and HCC recurrence were significantly associated with patient survival. On multivariate analysis, recipient age (HR, 1.06; 95% CI, 1.01–1.12; *p* = 0.031), use of bridging therapies (HR, 2.50; 95% CI, 1.12–5.55; *p* = 0.024), and HCC recurrence (HR, 2.58; 95% CI, 1.01–6.67; *p* = 0.048) remained significant predictors of patient survival. Other variables, including MELD score, hepatitis C virus (HCV) cirrhosis, alcoholic cirrhosis, AFP, downstaging therapy, and type of liver graft (uDCD/DBD), did not influence patient survival (Table 4).

**Table 4:** Univariate and multivariate analysis for predictors of patient survival.

Variables	HR	Univariate analysis		HR	Multivariate analysis	
		95%CI	*P* value		95%CI	*p* value
**Recipient**						
Age	1.05	1.01–1.10	*0.023*	1.06	1.01–1.12	*0.031*
MELD score	1.01	0.95–1.05	0.878			
HCV cirrhosis	1.28	0.62–2.63	0.492			
Alcoholic cirrhosis	0.56	0.26–1.23	0.148			
**HCC**						
Bridging therapy	2.87	1.31–6.28	*0.008*	2.5	1.12–5.55	*0.024*
AFP	0.99	0.96–1.01	0.576			
Downstaging therapy	1.11	0.45–2.16	0.877			
HCC Recurrence	4.41	1.89–10.27	*0.001*	2.58	1.01–6.67	*0.048*
**Graft**						
uDCD/BDD	1.28	0.67–2.42	0.441			

Italic values indicate statistically significant.

*AFP* alpha-fetoprotein; *CI* confidence interval; *DBD* donation after brain death; *HCC* hepatocellular carcinoma; *HCV* hepatitis C virus; *HR* hazard ratio; *MELD* model for end-stage liver disease; *OLT* orthotopic liver transplantation; *uDCD* uncontrolled donation after circulatory death.

On univariate analysis for predictors of HCC recurrence after transplantation, only use of TACE as therapy before OLT was significantly associated with recurrence (OR, 4.22; 95% CI, 1.15–20.06; *p* = 0.041). On multivariate Cox logistic regression analysis, TACE before OLT persisted as a significant risk factor for post-OLT tumor recurrence (OR, 3.93; 95% CI, 1.05–18.96; *p* = 0.045). Other variables did not influence HCC recurrence (Table 5).

**Table 5:** Univariate and multivariate analysis for predictors of hepatocellular recurrence after transplantation.

Variables	OR	Univariate analysis		OR	Multivariate analysis	
		95%CI	*p* value		95%CI	*p* value
uDCD graft	0.906	0.19–3.33	0.889			
**Bridging therapy**						
TACE	4.216	1.15–20.06	*0.041*	3.932	1.05–18.96	*0.045*
Radiofrequency	0.088	0.01–0.89	0.162	0.171	0.01–0.98	0.103
Within Milan criteria	0.636	0.09–12.65	0.689			
Differentiation	0.175	0.01–3.96	0.745			
mVi	0.362	0.06–20.46	1.500			
AFP at listing	0.979	0.89–1.00	0.475			
Tumor numbers	1.135	0.59–1.82	0.634			
Tumor size	1.203	0.77–1.78	0.373			
Waitlist time	0.997	0.99–1.00	0.351			

Italic values indicate statistically significant.

*AFP* alpha-fetoprotein; *CI* confidence interval; *mVi* microvascular invasion; *OR* odds ratio; *TACE* transarterial chemoembolization; *uDCD* uncontrolled donation after circulatory death.

## Discussion

Regarding the indications of OLT for HCC our OLT team criteria coincides with the Consensus Statement and Recommendations of the Spanish Society for Liver Transplantation. Thus, we are in complete agreement with this Society that accepts OLT for patients beyond Milan but within “up-to-seven” UCSF criteria with alpha-fetoprotein < 400 ng/ml and radiological response to locoregional therapy^24^. Prior reports using DBD livers identified several predictive factors for post-OLT HCC recurrence, such as advanced donor age^25,26^, diabetes mellitus, severe donor steatosis^18^, WIT > 50 min^17,27^, CIT > 10 h^27^, increased tumor size and number^20,21,25^, vascular invasion^27,28^, poor tumor differentiation^27,29^, elevated pre-OLT AFP, exceeding Milan criteria^27^, grafts from a non-local share distribution^21^, and unfavorable tumor biology on pre-OLT imaging^17^.

The use of selected cDCD donors offers excellent long-term graft and patient survival that are comparable to those observed with DBD donors, although ischemic cholangiopathy and subsequent BCs constitute specific morbidities associated with cDCD livers^30–33^. A recent multicenter study suggests that use of postmortem NRP in cDCD donors may reduce rates of post-OLT BCs and graft loss^34^. Several other OLT teams have reported using livers from cDCD donation in patients with HCC^16,32,35,36^, with the current tendency to use livers from cDCD donation rather than DBD livers in recipients with HCC^32,35^ and low MELD scores. The general impression is that these marginal grafts are better tolerated by recipients with better physical condition and liver function^37,38^. Thus, patients with HCC and low MELD scores are probably ideal candidates for cDCD livers^36,38^.

Preliminary studies from the Scientific Registry of Transplant Recipients demonstrated that use of cDCD livers increased the risk of death in OLT recipients with HCC^35^ and was associated with inferior patient and graft survival^16^. More recently, other OLT series comparing outcomes between cDCD and DBD livers in patients with HCC showed no differences in HCC recurrence^5,26,36^, patient survival, or tumor-free survival^5,36^. Furthermore, Khorsandi et al.^36^ found no difference in survival between cDCD and DBD transplant recipients but noted that increased HCC size and number, microvascular invasion, and poor tumor differentiation were associated with increased cancer-specific mortality, thereby highlighting the importance of tumor biology on survival and recurrence post-OLT in patients with HCC.

As with cDCD livers, uDCD livers have often been used in patients with HCC^7–11,39^, reaching up to 85–90% of patients in two recent series^9,10^ (one of which involved 46% of patients exceeding the Milan criteria)^9^. These series also underline the importance of using uDCD livers in recipients with HCC who meet recommended indications and avoiding their use when post-OLT graft function is unpredictable^9^. In agreement with previous authors^9^, our results confirmed that use of uDCD livers confers the advantage of reducing waitlist time and the dropout rate, compared with DBD livers. Similar to donor data of previously reported studies^7–10^, the mean age of our uDCD donors was significantly lower than DBD donors, yet the mean age of our uDCD recipients was significantly higher.

As in other cDCD donation^40,41^ and uDCD donation experiences^8–11,39,42^ the main disadvantages of uDCD livers in our series, when compared with DBD livers, were the increased need for blood products and higher rates of PNF, ischemic BCs, and retransplantation. PNF is a severe complication of OLT with a high mortality rate, given that early retransplantation in the only treatment available. Its occurrence has been related to a series of risk factors, including the use of grafts from extended criteria donors, hemodynamically unstable recipients, or those that require transfusion of large volumes of blood products. Regarding the use of uDCD livers, an increase in PNF has been linked to the longer period of ischemia. The incidence of PNF is 1.1–7.2% for DBD OLT, 1.8–11.8% when using cDCD livers, and 5–25% for uDCD OLT^22^. However, on the 30^th^ post-OLT day, liver function of our uDCD recipients was equivalent to that of DBD recipients, reflecting the good recovery of uDCD livers.

Overall, our results support the use of cDCD^6,43^ and uDCD livers^9–11^ to mitigate liver shortages, and mortality, especially in HCC patients. Because of better uDCD donor and recipient selection and management, our results have improved over the past 4 years, producing lower rates of PNF and BCs and better survival^11,44^.

Regarding pre-OLT HCC characteristics, the only significant difference between our groups was the shorter interval between HCC diagnosis and OLT in uDCD recipients. Tumor size, tumor number, AFP level, and percentage of patients within Milan or UCSF criteria were not significantly different between types of OLT. Similarly, there was no significant difference in number of patients who received pre-OLT bridging therapies. Despite use of bridging therapies in patients on the waitlist, tumor progression continued during this period (proportion of patients exceeding Milan criteria increased from 59.2% to 83.4% in the DBD group vs. 65.8–95.1% in the uDCD group). However, the overall HCC recurrence rate after OLT was similar in both groups (7.8% in DBD vs. 7.3% in uDCD), with parallel distributions of metastasis locations. Other authors have reported similar HCC recurrence rates between DBD and cDCD liver recipients (12.1% vs. 12.3%)^5^.

Previous studies reported no differences in 1-year patient survival between recipients of uDCD and DBD livers but lower 1-year graft survival in uDCD liver recipients^8,9,11,42^. Our study confirmed similar overall patient survival, disease-free survival, and tumor recurrence rates at 5 years between recipients receiving DBD and uDCD livers but lower 5-year graft survival in the uDCD group, which was mainly attributed to a higher incidence of ischemic BCs and PNF. The increased risk of these complications with cDCD^32,34^ or uDCD livers^7,9,10,39,44^ is well known, and when we excluded patients with PNF (the number of which has decreased substantially during the past 4 years) from our analysis, graft survival was not significantly different between groups.

Our multivariate analysis revealed recipient age, use of bridging therapies, and HCC recurrence as independent risk factors for patient survival, whereas pre-OLT bridging therapy with TACE was the only risk factor for HCC recurrence. The authors of a recent study found no association between locoregional therapy and worse patient survival, and they considered tumor biology (size, number, differentiation, and microvascular invasion) to be more relevant for patient survival^36^. Nevertheless, the need for bridging therapies may reflect unfavorable HCC biology and, therefore, a higher risk of recurrence and lower survival. Thus, bridging locoregional therapies for patients within Milan criteria do not improve post-OLT survival or recurrences in the majority of patients who fail to achieve complete pathologic response^45^. It has been suggested that delaying OLT for 3 months after inclusion on the waitlist can reduce early HCC recurrence by excluding patients with poor tumor biology^46^.

There are some limitations to this study. Data were collected retrospectively and were therefore subject to the typical biases expected with this study design. Although this study represents the largest single-center experience using uDCD livers in recipients with HCC, the sample size was limited for analyzing risk factors by multivariate analysis.

## Conclusion

In conclusion, when comparing uDCD and DBD livers in HCC recipients, similar patient survival but lower graft survival was observed in the uDCD group, which was attributed to the group’s higher incidence of PNF and BCs. Furthermore, HCC recurrence rates after OLT were similar with uDCD or DBD liver grafts, and TACE as a bridging therapy was identified as a risk factor for post-OLT recurrence. Other important advantages of using livers from uDCD in patients with HCC are the potential reduction in waiting list time, and consequently the decrease in the dropout rate of patients due to tumor progression or death.

## Data Availability

The dataset generated and analyzed for this study are available from the corresponding author on reasonable request.

## Abbreviations

ABS: Anastomotic biliary stricture
AFP: Alpha-fetoprotein
BC: Biliary complication
BMI: Body mass index
cDCD: Controlled donor after circulatory death
CI: Confidence interval
CIT: Cold ischemia time
CPR: Cardio-pulmonary resuscitation
DBD: Donation after brain death
DCD: Donor after circulatory death
DWIT: Donor warm ischemia time
FFP: Fresh frozen plasma
GGT: Gamma-glutamyl transpeptidase
GOT: Glutamic oxaloacetic transaminase
GPT: Glutamic pyruvic transaminase
HR: Hazard ratio
HBV: Hepatitis B virus
HCC: Hepatocellular carcinoma
HCV: Hepatitis C virus
ICU: Intensive care unit
INR: International normalized ratio
IRI: Ischemia/reperfusion injury
MELD: Model for end-stage liver disease
MMF: Mycophenolate mofetil
mTORi: Mammalian target of rapamycin inhibitors
NABS: Non-anastomotic biliary stricture
NRP: Normothermic regional perfusion
OLT: Orthotopic liver transplantation
OR: Odds ratio
PNF: Primary nonfunction
PRBC: Packed red blood cells
RWIT: Recipient warm ischemia time
TACE: Transarterial chemoembolization
uDCD: Uncontrolled donor after circulatory death
UCSF: University California, San Francisco
WIT: Warm ischemia time
